# Insights from DCE-MRI: blood–brain barrier permeability in the context of MS relapses and methylprednisolone treatment

**DOI:** 10.3389/fnins.2025.1546236

**Published:** 2025-03-20

**Authors:** Stig P. Cramer, Nizar Hamrouni, Helle J. Simonsen, Mark B. Vestergaard, Aravinthan Varatharaj, Ian Galea, Ulrich Lindberg, Jette Lautrup Frederiksen, Henrik B. W. Larsson

**Affiliations:** ^1^Department of Clinical Physiology and Nuclear Medicine, Copenhagen University Hospital – Rigshospitalet, Copenhagen, Denmark; ^2^Clinical Neurosciences, Clinical and Experimental Sciences, Faculty of Medicine, University of Southampton, Southampton, United Kingdom; ^3^Wessex Neurological Centre, University Hospital Southampton NHS Foundation Trust, Southampton, United Kingdom; ^4^Department of Neurology, Copenhagen University Hospital – Rigshospitalet, Copenhagen, Denmark; ^5^Department of Clinical Medicine, University of Copenhagen, Copenhagen, Denmark

**Keywords:** MRI, DCE MRI, perfusion MRI, blood–brain barrier, multiple sclerosis: multiple sclerosis relapses, methylprednisolone

## Abstract

**Background:**

Detecting multiple sclerosis (MS) relapses remains challenging due to symptom variability and confounding factors, such as flare-ups and infections. Methylprednisolone (MP) is used for severe relapses, decreasing the number of contrast-enhancing lesions on MRI. The influx constant (K_i_) derived from dynamic contrast-enhanced MRI (DCE-MRI), a marker of blood–brain barrier (BBB) permeability, has shown promise as a predictor of disease activity in relapsing–remitting MS (RRMS).

**Objectives:**

To investigate the predictive value of K_i_ in relation to clinical MS relapses and MP treatment, comparing its performance with traditional MRI markers.

**Methods:**

We studied 20 RRMS subjects admitted for possible relapse, using DCE-MRI on admission to assess K_i_ in normal-appearing white matter (NAWM) via the Patlak model. Mixed-effects modeling compared the predictive accuracy of K_i_, the presence of contrast-enhancing lesions (CEL), evidence of brain lesions (EBL; defined as the presence of CEL or new T2 lesions), and MP treatment on clinical relapse events. Five models were evaluated, including combinations of K_i_, CEL, EBL, and MP, to determine the most robust predictors of clinical relapse. Model performance was assessed using accuracy, sensitivity, specificity, positive predictive value (PPV), and negative predictive value (NPV), with bootstrapped confidence intervals.

**Results:**

Superior predictive accuracy was demonstrated with the inclusion of EBL and K_i_, alongside MP treatment (AIC = 66.12, *p* = 0.006), outperforming other models with a classification accuracy of 83% (CI: 73–92%), sensitivity of 78% (CI: 60–94%), and specificity of 86% (CI: 74–97%). This model showed the highest combined PPV (78%, CI: 60–94%) and NPV (86%, CI: 74–98%) compared to models with EBL or CEL alone, suggesting an added value of K_i_ in enhancing predictive reliability.

**Conclusion:**

These results support the use of K_i_ alongside conventional MRI imaging metrics, to improve clinical relapse prediction in RRMS. The findings underscore the utility of K_i_ as a marker of MS-related neuroinflammation, with potential for integration into relapse monitoring protocols. Further validation in larger cohorts is recommended to confirm the model’s generalizability and clinical application.

## Introduction

Multiple sclerosis (MS) is a chronic autoimmune disorder of the central nervous system characterized by inflammation, demyelination, and neurodegeneration. Relapses are the hallmark of the relapsing–remitting form (RRMS), and their detection is central to diagnosis and management. However, detecting clinical relapses can be challenging due to the wide range of presentations and confounding factors. One such factor is Uthoff’s phenomenon, which describes transient neurological symptoms triggered by factors such as heat exposure, overexertion, or simple infections (Thrower, 2009). While systemic infections may cause pseudo-relapses, evidence also suggests an association with true relapse activity (Correale et al., 2006; Thrower, 2009) and disease progression (Stuart et al., 2024), further complicating the identification of genuine relapses. Furthermore, early initiation of efficacious disease modifying treatment has been shown to improve long-term outcome in MS patients (Cobo-Calvo et al., 2023). In this context, accurate and timely identification of relapses is crucial for initiating appropriate treatment and monitoring disease activity and progression.

Methylprednisolone (MP), a synthetic glucocorticoid, is commonly used for symptomatic treatment of severe relapses (Myhr and Mellgren, 2009; Smets et al., 2017) and has been shown to reduce inflammation and the number of contrast-enhancing lesions on T1-weighted magnetic resonance imaging (MRI) scans (Sellebjerg et al., 2003). However, the impact of MP treatment on long-term clinical outcomes remains limited (Hommes et al., 1996; Myhr and Mellgren, 2009; Smets et al., 2017).

A recent retrospective study of 199 MS patients with acute relapse activity, investigated the correlation between symptoms of a clinical MS relapse and the anatomical location of new lesional activity, finding a clinical-radiological mismatch in 43% of cases. In these cases, the symptoms did not correspond to new MRI lesional activity in the relevant anatomical brain region. Furthermore, in 33% of all cases, no new MRI lesional activity could be identified on brain or spinal cord MRI (Dünschede et al., 2023). This clinico-radiological paradox emphasizes the need for biomarkers capable of detecting clinical disease activity (Galea et al., 2015), particularly to guide decisions regarding symptomatic MP treatment of acute relapses and the escalation of disease-modifying therapies (DMT). While serum-derived biomarkers—especially neurofilament light chain - have shown considerable promise in monitoring disease activity and treatment response, findings on their ability to predict relapses remain conflicting (Højsgaard Chow et al., 2024). Therefore, imaging-derived biomarkers may still hold potential for future benefit.

Dynamic contrast-enhanced magnetic resonance imaging (DCE-MRI) is a non-invasive imaging technique that allows the measurement of blood–brain barrier (BBB) permeability (Larsson et al., 1990; Sourbron et al., 2009). The influx constant, K_i_, derived from DCE-MRI data, reflects the rate at which the contrast agent leaks into the brain parenchyma and can serve as an imaging marker of subtle changes in BBB integrity (Larsson et al., 2009). Previous studies have demonstrated the predictive capabilities of K_i_ in the context of conversion from optic neuritis to MS (Cramer et al., 2015) and in monitoring treatment effects in the normal-appearing white matter (NAWM) away from MS lesions (Cramer et al., 2018; Knudsen et al., 2022).

Given the potential of K_i_ as a sensitive marker of neuroinflammation, the present study aimed to investigate the impact of acute MS relapses and MP treatment on K_i_. By prospectively analyzing a cohort of patients with relapsing–remitting MS (RRMS) suspected of acute relapse activity, we aim to elucidate the development of K_i_ in individual subjects, during relapse and MP treatment response. Additionally, we aimed to explore the relationship between K_i_ and conventional MRI markers, such as MRI contrast-enhancing lesions and new T2 lesions.

## Methods

### Study participants

**Figure 1:**
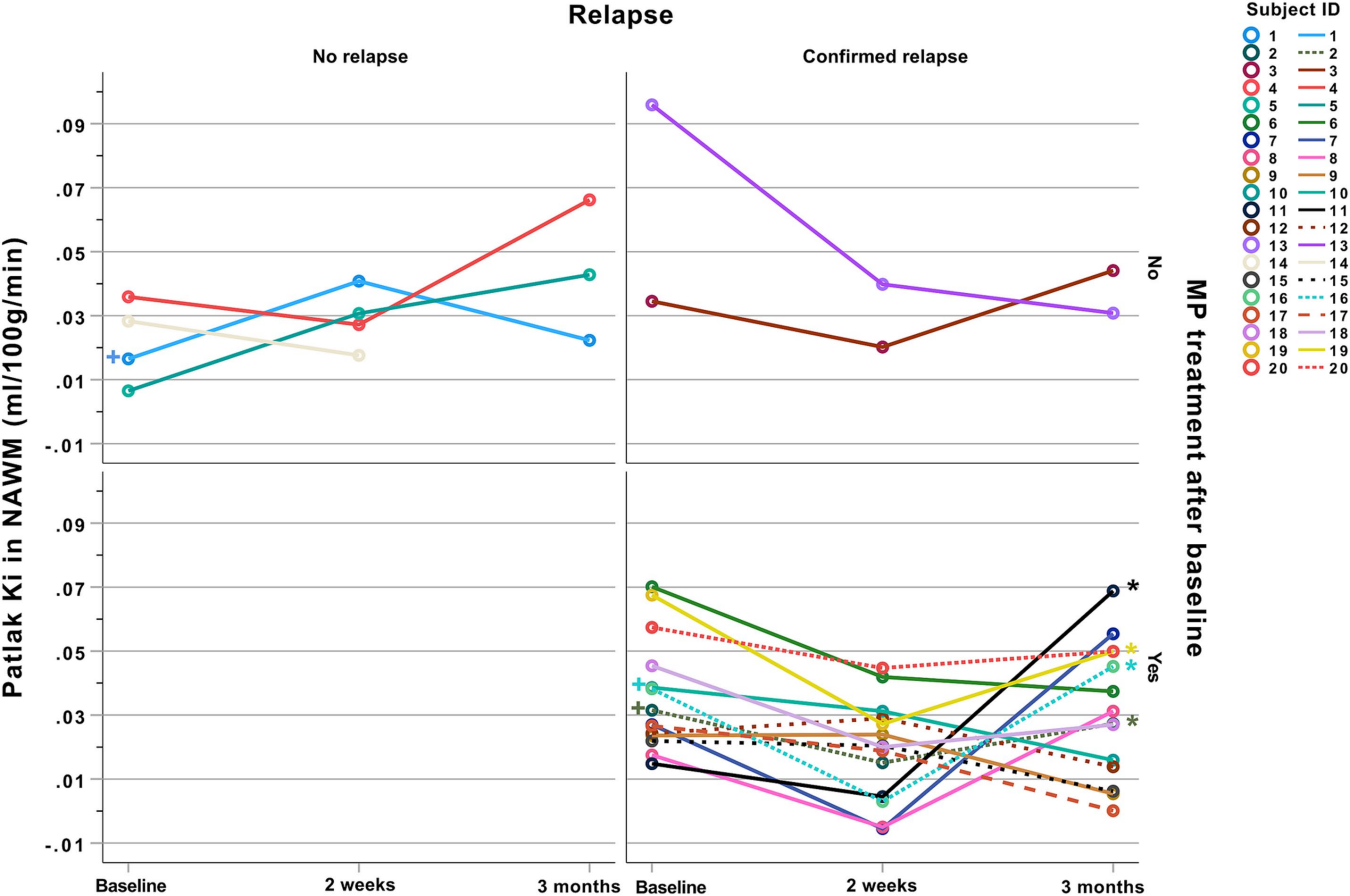
Study timeline. Twenty subjects with RRMS suspected of an acute MS relapse were included in the study and MRI scanned at baseline, and 2 weeks after MP treatment (when relevant from a clinical perspective) and again 3 months post-relapse. MP, Methylprednisolone.

Twenty patients with established relapsing remitting MS (RRMS) were referred from the Department of Neurology Rigshospitalet, Glostrup, suspected of a MS relapse. The inclusion criteria were an established diagnosis of RRMS, clinical suspicion of relapse and willingness to participate in three consecutive scanning sessions. All subjects referred to the study were included. MRI scans were performed at time of study inclusion, again after 2 weeks, and the final scan was performed as a post-relapse scan after 3–4 months. For study timeline overview see Figure 1.

Clinical MS relapse was defined as the development of a new symptom, or worsening of a pre-existing symptom, confirmed on neurologic examination, lasting at least 48 h, and preceded by stability or improvement lasting at least 30 days. Neurologic deterioration only temporarily associated with a period of fever was not considered a relapse (Schumacker et al., 1965). Relapses were categorized according to severity into major and minor relapses. Major relapses had an increase of >1 point on the Expanded Disability Status Scale (EDSS) (>0.5 points above EDSS 5.5) since the preceding visit. A minor relapse was defined as an exacerbation with an increase of >1 point in any of the functional system sub scores but not leading to a change in EDSS (Buljevac et al., 2002). Infections were defined as the presence of coryza, sore throat, flu-like symptoms, myalgia, fever, urinary infection, or diarrhea lasting more than 24 h. Urinary infections were defined as the presence of fever, chills, dysuria, increased need to void, suprapubic pain, and pathologic urine analysis or positive culture. Additionally, separate relapse activity occurring in proximity to any follow-up scan was defined as a new relapse event potentially influencing the outcome measures if occurring +/− 2 months before or after the scan date. MP treatment consisted of an oral or intravenous dose of 1 gram of methylprednisolone given once per day for three consecutive days (no tapering), initiated immediately after the baseline scan. Relapse classification and MP treatment decisions were made independently of the study by an experienced MS specialist consultant neurologist, who was blinded to the results of the DCE scan.

### Ethics

This study was approved by the Ethics Committee of Copenhagen County according to the standards of The National Committee on Health Research Ethics, protocol number H-D-2008-002. All experiments were conducted in accordance with the Helsinki Declaration of 1975 and all subjects gave written informed consent.

### Dynamic contrast-enhanced (DCE) MRI

MRI was performed on a 3T MR unit (Philips Achieva) using a 32-element phased-array head coil. DCE-MRI used a T1-weighted saturation-recovery gradient-echo sequence with flip angle 30°, repetition time = 3.9 ms, echo time = 1.9 ms, centric phase ordering, parallel imaging factor 2, acquired matrix 96 × 61, acquired voxel-size 2.40 × 2.98 × 8 mm^3^ (interpolated to 0.90 × 0.89 × 8 mm^3^), field of view 230 × 182 mm^2^, five slices, slice thickness 8 mm (Supplementary Figure 1). Data for an initial measurement of relaxation time (T_1_) and equilibrium magnetization (M_0_) were generated using a series of saturation time delays from 120 ms to 10 s, covering the same slices as imaged during the bolus passage. The dynamic sequence used a saturation time delay of 120 ms, giving a time resolution of 1.25 s, and 750 time points, corresponding to a total sampling duration of 15.7 min. The dose of contrast agent (Gadobutrol 1 mmoL/mL) was 0.09 mmol/kg bodyweight, injected automatically (Spectris, Medrad; USA) with a speed of 3 mL/s followed by 20 mL saline, injected as two boli of each 0.045 mmol/kg bodyweight at time points 15 and 80, corresponding to 18.8 and 100 s. We acquired a separate slice at the level of the internal carotid artery (ICA) to obtain an arterial input function with minimal partial volume for every single subject. The remaining four DCE slices were used for defining regions of interest and subsequent estimation of tissue pharmacokinetic values.

### MRI sequences and regions of interest

We used an axial T2-weighted MRI sequence [five slices, echo time = 100 ms, repetition time = 3000 ms, acquired voxel-size 0.57 × 0.76 × 8 mm^3^ (interpolated to 0.45 × 0.45 × 8 mm^3^), field of view = 230 × 119 mm^2^] with same orientation and slice thickness (8 mm) as our DCE-MRI sequence, in order to manually draw regions of interest in the frontal white matter, and the thalamic grey matter in both hemispheres. Four regions of interest (ROIs) were placed in frontal white matter (two in the vicinity of the frontal ventricular horns, one in each hemisphere and two in the vicinity of the posterior horn, one in each hemisphere). Two ROIs were placed in the right and left thalamus. The DCE slices and ROIs were placed with the same angulation and anatomical position on consecutive scans (Supplementary Figure 1). We ensured consistent positioning and size of our ROIs across study time points by visual alignment with the previous scan.

### Estimation of K_i_, cerebral blood volume (CBV) and cerebral blood flow (CBF)

The DCE MRI data was analysed with a semi-automated procedure (Larsson et al., 2008, 2009) using in-house Matlab-based software. The DCE time series was converted to units of contrast agent concentration using T_1_ and M_0_, as determined from the multiple saturation delay data, and a contrast agent relaxivity of 4 s^−1^ mM^−1^. The input function was measured in the voxel of the internal carotid artery with maximal signal change during the bolus passage and was corrected for partial volume by normalizing to a magnitude-and phase-derived venous outflow function, sampled in the sagittal sinus (Cramer et al., 2022). The mean signal-time curve for all voxels in the ROI was extracted and used to calculate permeability. Every subject was represented by one K_i_, CBV and CBF value, calculated as the mean of the tissue specific ROIs, as previously described (Cramer and Larsson, 2014; Cramer et al., 2014, 2015, 2022). Tissue concentration-time curves were evaluated using a combination of model-free deconvolution, yielding CBF, and a Patlak model, yielding CBV and K_i_, as described in previous work (Larsson et al., 2009). Permeability values, measured as K_i_ (full blood) relates to K^trans^ (plasma) by K_i_ = K^trans^/(1-Hct). A fixed value of Hct = 0.45 was used throughout the study. Values of K_i_ are reported as ml/100 g/min assuming brain tissue density of 1 g/mL (Barber et al., 1970).

### Statistics

Histograms, probability plots and modified Kolmogorov–Smirnov (Lillefors) testing were used to analyze continuous variables for standard normal distribution fit (Lilliefors, 1967; Dallal and Wilkinson, 1986). For comparisons between continuous variables Wilcoxon signed-rank tests were performed and for categorical data Chi square tests were performed. For comparisons between more than two groups, ANOVA testing was used. To predict clinical relapse (binary variable; y/n), we evaluated the predictive accuracy of five mixed-effects models, each incorporating combinations of the following predictors: (1) Presence of contrast-enhancing lesions (CEL): A binary variable indicating the presence or absence of contrast-enhancing lesions. (2) “Evidence of brain lesion” (EBL), defined as either the presence of either CEL or new T2 lesions(s) when compared to a previous MRI. (3) K_i_ in normal-appearing white matter. (4) MP use (y/n) was added as a confounder to account for treatment effects, given its known influence on blood–brain barrier integrity. Finally, a random intercept for patient ID (subj) was added to account for repeated measurements within subjects.

The five models evaluated were:

**Table:** 

Model 1	Relapse ~ K_i_ + MP + (1 | subj)
Model 2	Relapse ~ EBL + MP + (1 | subj)
Model 3	Relapse ~ CEL + MP + (1 | subj)
Model 4	Relapse ~ K_i_ + EBL + MP + (1 | subj)
Model 5	Relapse ~ K_i_ + CEL + MP + (1 | subj)

We used the Akaike Information Criterion (AIC), the Bayesian Information Criterion (BIC), and likelihood ratio tests to compare model fits. These metrics allowed us to evaluate both model simplicity (AIC/BIC) and model improvement through nested comparisons (Chi-squared tests). Once the best model was selected, we further evaluated model performance using key classification metrics: Accuracy: proportion of correct predictions. Sensitivity: proportion of actual positive cases correctly identified. Specificity: proportion of actual negative cases correctly identified. Positive Predictive Value (PPV) and Negative Predictive Value (NPV): indicating the predictive accuracy for positive and negative relapse cases. Bootstrapping was applied to estimate confidence intervals around these metrics, providing robust measures of variability. To quantify the effect of relapse (y/n) and MP treatment (y/n) on K_i_ we performed a *post-hoc* mixed model general linear regression analysis with K_i_ as the dependent variable and relapse and MP treatment as explanatory variables. A *p*-value lower than 0.05 allowed rejection of the null hypothesis. Statistical analysis was performed using SPSS 28.0, with the assistance of a professional statistician from the Biostatistical Department at the University of Copenhagen.

## Results

### Data attrition

Nineteen out of 20 subjects completed all three scanning sessions, while one subject (subj 14) declined to participate in the final three-month scan, giving a total of 59 scanning sessions with complete structural MRI and K_i_ measurement data. At study entry 16 out of 20 subjects (80%) were classified as having a clinical MS relapse at study entry, of which 14 received MP treatment. Of the 16 with a relapse, six were classified as having a major relapse (38%). Three subjects had received MP treatment within 6 weeks before study entry due to a previous relapse. During the follow-up period, four subjects were classified as having a new, separate relapse, three of which required a course of MP treatment. Subject demographics and clinical variables are shown in Table 1. Clinical description of the relapse symptoms, MP use pre-and post-baseline, and information on relapses during the follow-up period for each subject is described in Table 2. T1 contrast-enhancing lesions were present at baseline in eight out of 20 subjects (40%). Ten subjects (50%) had new T_2_ white matter lesions at baseline when compared to the most recent previous MRI scan, and eight out of 10 had MRI contrast enhancement in one or more of these new T2 lesions.

**Table 1:** Subject demographics, clinical data, and K_i_ values according to relapse status at baseline.

			Relapse status	
			No relapse (*n* = 4)	Confirmed relapse (*n* = 16)
Age (years)	Mean (SD)		38 (10)	34 (11)
EDSS	Median (IQR)		2.3 (2)	2.3 (2.3)
Symptom onset to MRI (days)	Median (IQR)		12 (10)	6 (11)
Gender	Female	*n* (%)	3 (75%)	14 (88%)
	Male	*n* (%)	1 (25%)	2 (12%)
MP use 6 weeks prestudy	No	*n* (%)	3 (75%)	14 (88%)
	Yes	*n* (%)	1 (25%)	2 (12%)
MP treatment after baseline	No	*n* (%)	4 (100%)	2 (12%)
	Yes	*n* (%)	0 (0%)	14 (88%)
Disease modifying treatment	None	*n* (%)	1 (25%)	5 (31%)
	First-line	*n* (%)	1 (25%)	4 (25%)
	Second-line	*n* (%)	2 (50%)	7 (44%)
New T2 lesions at baseline	0	*n* (%)	3 (75%)	7 (44%)
	≥1	*n* (%)	1 (25%)	9 (56%)
Contrast-enhancing lesions at baseline	0	*n* (%)	3 (75%)	7 (44%)
	≥1	*n* (%)	1 (25%)	9 (56%)
CEL volume at baseline (mm^3^)	Median (range)		0 (0–509)	0 (0–654)
Baseline Patlak K_i_ in NAWM (ml/100 g/min)	Mean (SD)		0.022 (0.013)	0.040 (0.022)
2 weeks Patlak K_i_ in NAWM (ml/100 g/min)	Mean (SD)		0.029 (0.010)	0.021 (0.015)
3 months Patlak K_i_ in NAWM (ml/100 g/min)	Mean (SD)		0.044 (0.022)	0.033 (0.022)

EDSS, Expanded Disability Status Scale; MP, Methylprednisolone; CEL, contrast-enhancing lesion(s); NAWM, Normal appearing white matter.

**Table 2:** Clinical characteristics of individual relapses.

Subject	Duration of MS (months)	EDSS	Clinical symptoms and signs	Days from symptom onset to MRI	Baseline MRI	Clinical relapse	MP treatment after baseline scan	Baseline DMT	Pre baseline MP	No. of additional confirmed relapses**
1	0	0.0	Increased fatigue	10	1 new large “tumefactive” T2 lesion in the left hemisphere with edema; 3 CELs	No*	No	None (NTZ after two-week scan)	Yes; 20 days prior	0
2	1	2.0	Left hemi sensory loss, diplopia, loss of balance, dysarthria	6	7 new T2 lesions, 20 CELs	Yes (major)	Yes	None	Yes; 30 days prior	1 (27 days after 3 months scan)
3	24	2.5	Allodynia right LE	3	Status quo	Yes (minor)	No	IFN-*β*	No	0
4	65	2.5	Awoken with transient aphasia suspicious of TCI; history of stroke and MS	0	Status quo	No (TIA final diagnosis)	No	DMF	No	0
5	60	2.5	Slight vertigo and fatigue	11	Status quo	No*	No	NTZ	No	0
6	10	1.0	Muscle weakness and ataxia in right UE. Increased fatigue.	8	Status quo	Yes (major)	Yes	TFL	No	0
7	41	1.5	Progression over days of reduced muscle strength and sensory loss bilateral LE, and increase in bladder dysfunction.	18	2 new T2 lesions, that are both CEL	Yes (major)	Yes	None	No	0
8	0	1.5	Muscle weakness (4/4+) and sensory loss in left LE and UE	6	Status quo	Yes (major)	Yes	None	No	0
9	46	4.0	Right hemisensory loss, dysarthria and loss of balance	4	Multiple (>10) new T2 lesions; 10 CELs	Yes (major)	Yes	FTY	No	0
10	0	0.0	Sensory loss right LE (14 days), and pain in left eye on lateral gaze	5	One new T2 that is also a CEL	Yes (minor)	Yes	None	No	0
11	3	3.0	Sensory loss left UE, vertigo, nausea, headache	3	Five new T2 lesions, of these 2 are CEL	Yes (minor)	Yes	TFL	No	Yes (49 days after 3 months scan)
12	24	2.0	Sensory loss left hand, loss of balance, vertigo, nausea	3	Status quo	Yes (minor)	Yes	FTY	No	0
13	60	2.5	Muscle weakness and sensory loss in right UE	3	Status quo	Yes (minor)	No	IFN-*β*	No	0
14	13	2.0	Increased fatigue	14	Status quo	No*	No	NTZ	No	0
15	14	2.5	Left hemi-body allodynia and hyperesthesia	13	4 new T2 lesions	Yes (minor)	Yes	FTY	No	0
16	0	1.0	Vertigo of relatively sudden onset	5	6 new supratentorial T2 lesions and 1 new lesion in pons; multiple CELs	Yes (minor)	Yes	None	Yes; 25 days prior	2 (76 days *before* 3 months scan, and 58 days after)
17	111	0.0	Left hemifield visual defect, diplopia and severe nausea	3	One new T2 lesion that is also a CEL	Yes (major)	Yes	FTY	No	0
18	120	3.5	Worsening in gait over four weeks, reduced hip and knee muscle strength (4+) and foot drop on both sides	25	Status quo	Yes (major)	Yes	RTX	No	0
19	108	4.0	Post-partum gradual onset ataxia right UE, balance loss, right visual hemifield defect and markedly reduced walking distance	29	1 new T2 lesion (juxtacortical)	Yes (major)	Yes	NTZ	No	1 (49 days after 3 months scan)
20	60	0.0	Awoken with sensory loss left LE progressing during the day, later same day onset of reduced muscle strength left LE.	8	Status quo	Yes (major)	Yes	NTZ	No	0

*Purely subjective symptoms not classified as a relapse by the clinician. **Additional separate relapses (not related to the relapse at study inclusion) occurring during the study period and up to 3 months after the final scan date. ALZ, alemtuzumab; CEL, contrast-enhancing lesion; DMF, dimethyl fumarate; DMT, disease-modifying therapy; EDSS, Expanded Disability Status Scale; FTY, fingolimod; GA, glatiramer acetate; IFN-*β*, *β*-interferons; LE, Lower Extremity; NTZ, natalizumab; OCR, ocrelizumab; RTX, rituximab; TFL, teriflunomide; TIA, transient ischemic attack; UE, Upper Extremity.

### Relapse prediction

**Figure 2:**
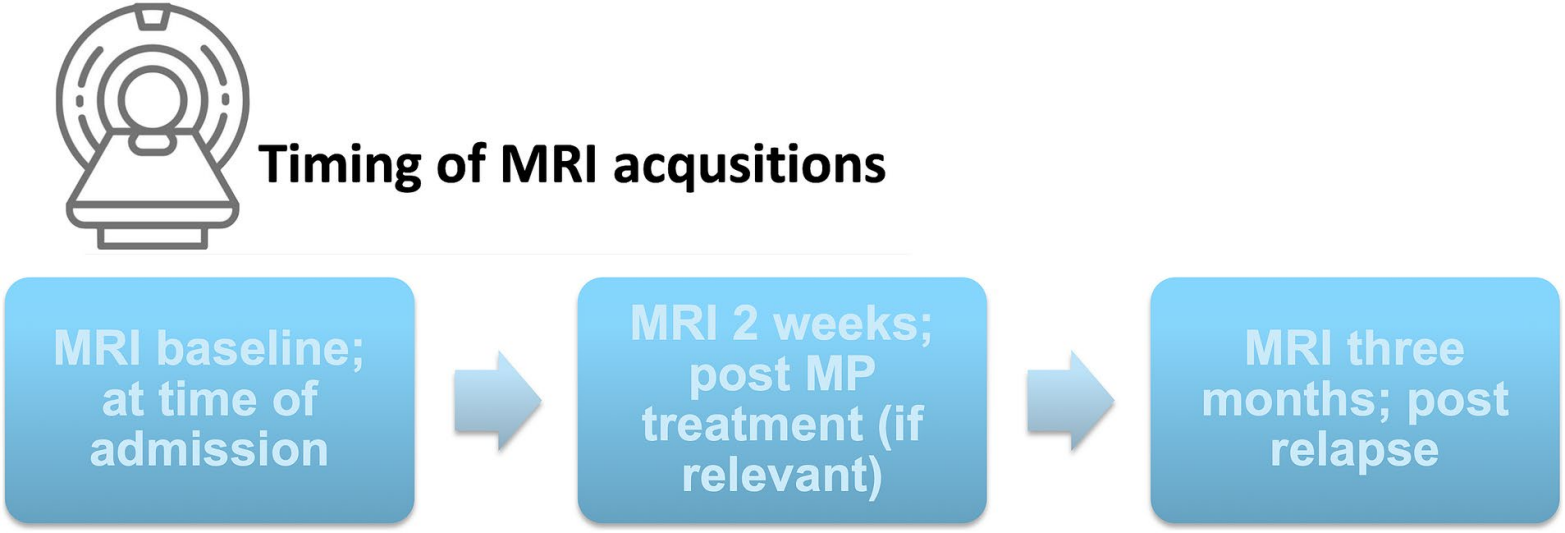
Spaghetti plots of K_i_ development during the study. Influx constant (K_i_) in NAWM for each subject plotted over time divided by confirmed relapse and MP treatment. A *post-hoc* mixed model general linear regression analysis showed that K_i_ was 0.011 mL/100 g/min higher (*p* = 0.03) in the context of relapse and 0.018 mL/100 g/min lower (*p* = 0.002) after MP use. Four patients were classified as having a new relapse within +/− 2 months from the post-relapse months scan, marked with (*). Three subjects received MP treatment <6 weeks prior to study inclusion, marked with (+). MP, Methylprednisolone; NAWM, Normal appearing white matter.

**Figure 3:**
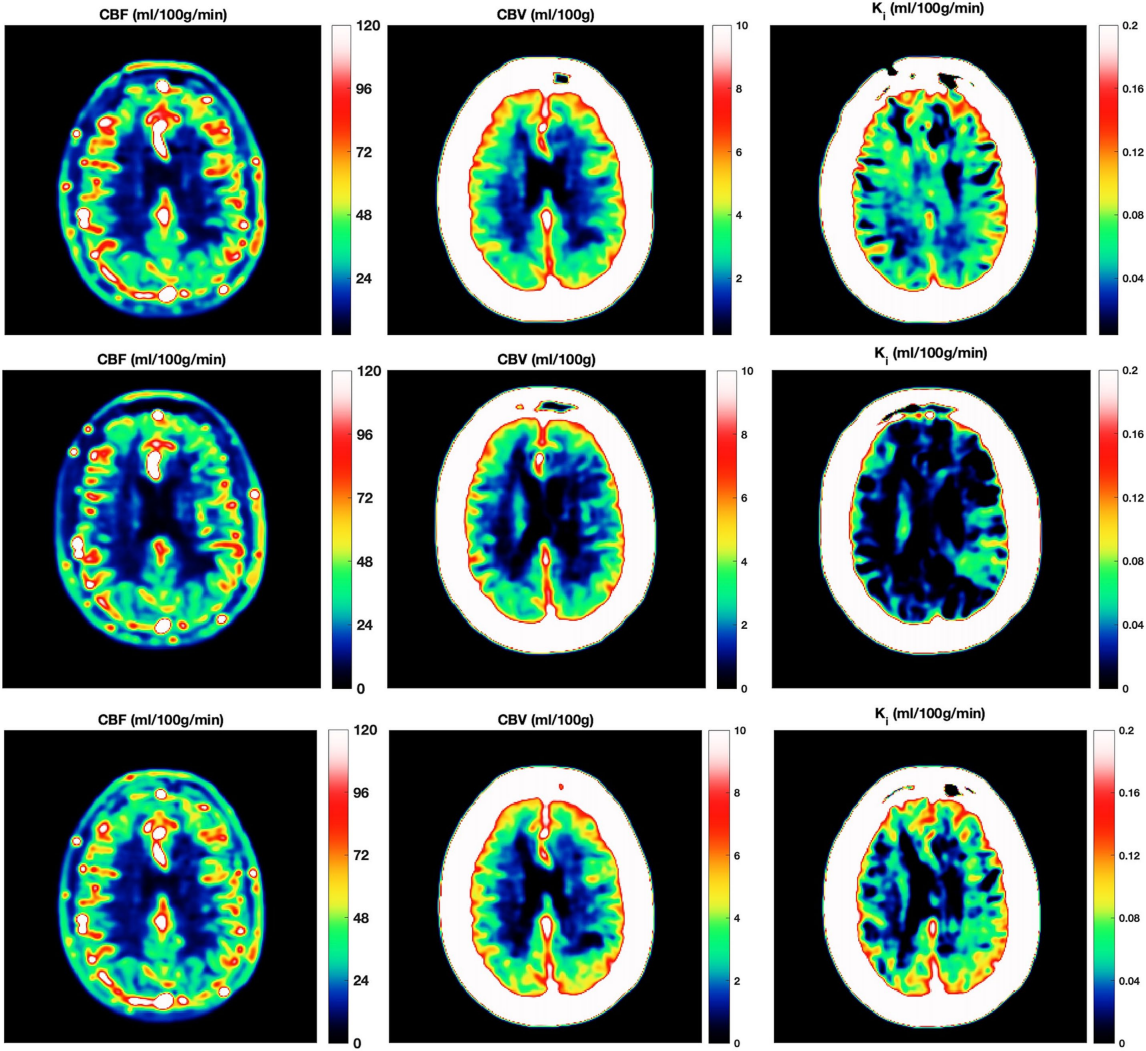
Kinetic parameter maps. Three serial DCE-MRI scans of the same RRMS subject with a confirmed relapse (top row = at relapse onset; middle row = 2 weeks post MP treatment; bottom row = 3 months post relapse). Voxel wise cerebral blood flow (CBF; left column), cerebral blood volume (CBV; middle column) and Influx constant (K_i_; right column) from one DCE slice. Note that CBF and CBV are unchanged, while K_i_ is initially high, low after 2 weeks of MP treatment, and increases again after 3 months. CBF, cerebral blood flow; CBV, cerebral blood volume; DCE-MRI, dynamic contrast-enhanced MRI; K_i_, Influx constant; MP, Methylprednisolone.

**Figure 4:**
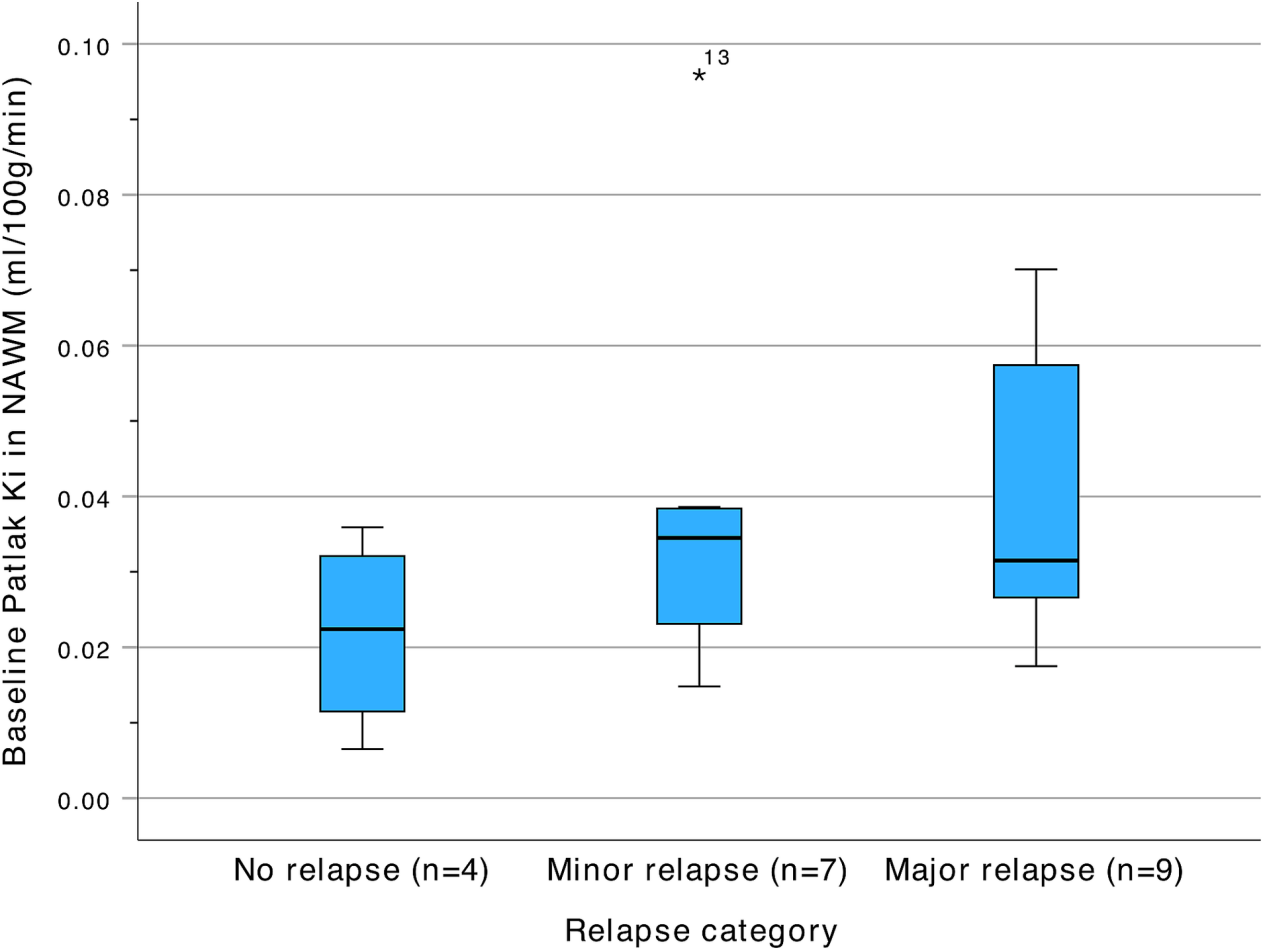
Baseline Patlak K_i_ in NAWM according to relapse severity at time of admission. NAWM, Normal appearing white matter.

We evaluated the five constructed models on key classification metrics—accuracy, sensitivity, specificity, PPV, and NPV— using bootstrapping for confidence intervals. Since CEL and EBL showed strong colinearity we avoided adding them simultaneously to the same model. Model performance metrics and confidence intervals for the investigated models are depicted in Table 3. Model 4 [Relapse ~ K_i_ + EBL + MP use + (1 | subj)], demonstrated the best fit based on the AIC, BIC, and Chi-squared tests showing the lowest deviance and a significant improvement over the other models (*p* = 0.006). Regarding diagnostic accuracy Model 4 achieved the highest combined accuracy and PPV, at the expense of a slightly decreased sensitivity and NPV, suggesting that including K_i_ along with EBL enhances predictive accuracy. The development of K_i_ during the study according to relapse and MP treatment status is shown in Figure 2. An example of the development of voxel-wise K_i_, CBV, and CBF from a representative subject with a major relapse can be seen in Figure 3. When further stratifying the subjects according to minor and major relapse, K_i_ did not significantly predict relapse severity but showed a trend for higher values in the major relapse group (see Figure 4). *Post-hoc* mixed model analysis showed a significant effect of relapse (*β* = 0.011, *p* = 0.03) and MP use (*β* = −0.018, *p* = 0.002) on K_i_, meaning that K_i_ was 0.011 mL/100 g/min higher in the context of relapse and 0.018 mL/100 g/min lower in the context of MP treatment.

**Table 3:** Mixed model performance metrics with bootstrapped confidence intervals.

Model	Metric	Estimate	95% CI
Model 1 (K_i_)	Accuracy	0.76	0.64–0.86
	Sensitivity	0.78	0.60–0.95
	Specificity	0.75	0.59–0.89
	PPV	0.67	0.48–0.85
	NPV	0.84	0.70–0.97
Model 2 (EBL)	Accuracy	0.78	0.68–0.88
	Sensitivity	0.91	0.78–1.00
	Specificity	0.69	0.54–0.84
	PPV	0.66	0.49–0.81
	NPV	0.93	0.81–1.00
Model 3 (CEL)	Accuracy	0.78	0.68–0.88
	Sensitivity	0.91	0.78–1.00
	Specificity	0.69	0.54–0.84
	PPV	0.66	0.48–0.81
	NPV	0.93	0.81–1.00
Model 4 (K_i_ + EBL)	Accuracy	0.83	0.73–0.92
	Sensitivity	0.78	0.60–0.94
	Specificity	0.86	0.74–0.97
	PPV	0.78	0.60–0.94
	NPV	0.86	0.74–0.97
Model 5 (K_i_ + CEL)	Accuracy	0.78	0.66–0.88
	Sensitivity	0.70	0.50–0.88
	Specificity	0.83	0.71–0.94
	PPV	0.73	0.53–0.90
	NPV	0.81	0.68–0.92

K_i_, influx constant derived from Patlak; EBL, Evidence of brain lesion; CEL, Contrast enhancing lesions; PPV, positive predictive value; NPV, negative predictive value; CI, Confidence interval.

### Conventional contrast imaging and K_i_

Eight out of 20 subjects (~40%) had one or more contrast-enhancing lesions on baseline MRI. When addressing MRI contrast-enhancing lesions in terms of both presence of 1 or more lesions (Supplementary Figure 2), or total lesion volume (Supplementary Figure 3), we did not observe any associations with K_i_ in neither NAWM nor in the thalamus. Nor did we find any significant associations between K_i_ and the presence of new T2 lesions or the total T2 lesion volume (ml).

## Discussion

### Relationship between relapses and imaging metrics

The best performance for predicting clinical relapses was achieved by adding a combination of conventional MRI metrics (contrast-enhancing lesions and new T2 lesions) as well as K_i_ in NAWM into the model, with a significant improvement in predictive performance compared to the other investigated models.

The strong performance metrics, including high sensitivity and specificity, indicate that the proposed model could serve as an effective tool in clinical settings, offering a balanced predictive ability for both positive and negative relapse cases. The inclusion of MP use as a confounder was essential to account for its impact on the blood–brain barrier and potential alterations in K_i_ values, underscoring the importance of controlling for clinical interventions in predictive modeling.

The relationship between K_i_, MS relapses, and MP use observed in this study is consistent with previous research showing a similar association but in a cross-sectional study design (Cramer et al., 2014). Thus, here we can substantiate this observation in a serial study design, indicating that K_i_ may reflect changes on the individual subject level, related to disease activity and treatment. The proposed model outperformed the other investigated models in terms of accuracy and PPV, suggesting that both K_i_ and EBL capture relevant but distinct aspects of the underlying MS pathophysiology, making their combined inclusion valuable. Regarding diagnostic accuracy Model 4 (K_i_ + EBL) achieved the highest combined accuracy and PPV, at the expense of a slightly decreased sensitivity and NPV, suggesting that including K_i_ along with EBL enhances predictive accuracy. Although the predictive values of both Model 4 and Model 5 offer clinical insights, Model 4 stands out with a higher Positive Predictive Value (PPV), meaning it more reliably identifies true attacks when positive results are indicated. This is clinically valuable, as it minimizes the risk of missing cases that may require follow-up, treatment and intervention. Therefore, Model 4 is preferred, as it provides a balance of accuracy and clinical relevance, increasing the likelihood that detected relapses accurately reflect a patient’s condition.

Notably, models incorporating only a single predictor (EBL, CEL, or K_i_) demonstrated inferior performance, underscoring the limitations of individual predictors in capturing the multifaceted clinical profile of MS relapses. For the conventional MR imaging metrics, this finding aligns with prior research reporting a modest yet statistically significant correlation with clinical relapses in RRMS over a short-term observation period, with a high sensitivity but relatively lower specificity (Kappos et al., 1999; Rovaris et al., 2003). It is perhaps noteworthy that the addition of K_i_ to the predictive model appears to improve specificity for relapse detection, thus providing unique and complementary information on the dynamics of subtle changes in MS inflammation beyond visible lesion activity.

A large degree of heterogeneity in K_i_ at the post-relapse scan was observed, where not all subjects exhibited the expected decrease in K_i_ despite being free of clinical relapse symptoms. This may have several possible interpretations, including, but not limited to (1) the effect of a relapse on K_i_ is delayed or lasts longer than 3 months, (2) subjects with an intrinsic aggressive MS phenotype show a persistently elevated K_i_ irrespective of current relapse activity, or (3) subjects currently receiving a suboptimal disease-modifying treatment have persistently elevated K_i_. To investigate this question, additional follow-up scans, or ideally a pre-relapse baseline scan, would have been of benefit.

### Impact of methylprednisolone (MP) treatment on K_i_ values

While it is well accepted that MP treatment reverses contrast-enhancement (Barkhof et al., 1991; Sellebjerg et al., 2003) and therefore large focal changes in BBB permeability, it has not yet been confirmed that this is the case with more widespread low-level BBB permeability change. Animal and human studies suggest that MP restores BBB integrity (Kesselring et al., 1989; Barkhof et al., 1991; Sellebjerg et al., 2003; Smets et al., 2017) while we have previously demonstrated a cross-sectional association between recent MP treatment and a less permeable blood–brain barrier, as measured by K_i_, indicative of a therapeutic effect (Cramer et al., 2014, 2018). The results presented here confirm that MP also reverses more widespread low-level BBB leakage, as measured with K_i_. This is in line with animal and human studies showing that MP exerts its therapeutic effects by suppressing immune cell infiltration (Myhr and Mellgren, 2009; Smets et al., 2017) and restoring BBB integrity (Kesselring et al., 1989; Barkhof et al., 1991; Sellebjerg et al., 2003; Smets et al., 2017). While MP treatment was significantly associated with K_i_ in the *post-hoc* mixed model, there was heterogeneity in the treatment response among patients. Some individuals exhibited a complete normalization of K_i_ values, with values close to those observed in healthy controls (Cramer et al., 2014; Varatharaj et al., 2019) while others showed only partial improvement. These variations in treatment response may reflect different trajectories in treatment response with respect to the extent of BBB disruption and baseline neuroinflammatory activity.

### Study limitations

The repeated measures approach used in this study yielded a total of 59 measurement points for K_i_ as well as for structural MRI data. While this should give sufficient power for performing a model analysis with three predictors, a key limitation of this study is still the relatively limited sample size, of 20 subjects, which may affect the generalizability of our findings. A smaller sample can increase the likelihood of overfitting, especially in models with multiple predictors. This limitation restricts our ability to make broad conclusions about the predictive accuracy of K_i_ in diverse populations and across different clinical settings. Additionally, while we included MP use as a confounder to control for its effects, other unmeasured confounders may also influence the outcome variable potentially biasing our results. Moreover, since our data were collected from a specific clinical population with unique characteristics, the predictive performance of the proposed model may vary when applied to a broader cohort or in patients with different comorbidities or clinical histories. Future research should validate this model across larger, more diverse populations to assess its robustness and establish whether these findings hold true in different patient groups.

A potential confounder regarding the “outside-of-relapse” scan is the influence of DMT initiation during the study period. Six subjects started new DMT or changed DMT shortly before the three-month follow-up, which may have influenced the K_i_ values. However, as previously reported, the effect of DMT on K_i_ appears to be negligible after 3 months of treatment, with a significant effect observed only after 6 months (Cramer et al., 2016) indicating that any confounding impact is likely minimal.

## Conclusion

In conclusion, this study identifies a robust predictive model for detecting MS relapses, that combines conventional MRI lesional information with K_i_ in NAWM, derived from DCE-MRI. This suggests that adding DCE-MRI could improve reliability in early identification of MS relapses. Future studies should consider validating this model in independent datasets and exploring additional predictors that may further improve prediction accuracy.

## Data Availability

The raw data supporting the conclusions of this article will be made available by the authors, without undue reservation.

## Supplementary material

SUPPLEMENTARY FIGURE 1 DCE-MRI overview. Dynamic contrast-enhanced MRI at 3T; 5 slices; 8 mm slice thickness; time resolution 1.25 s, total scan time 15 minutes.

SUPPLEMENTARY FIGURE 2 K_i_ according to presence on one or more contrast enhancing lesion. No difference in K_i_ in NAWM was observed in subjects with visually contrast enhancing lesions elsewhere in the cerebrum. NAWM, Normal appearing white matter.

SUPPLEMENTARY FIGURE 3 K_i_ versus total contrast enhancing lesions volume. Patlak K_i_ in NAWM plotted against the total volume of visual contrast enhancing lesions in ml. MP, Methylprednisolone; NAWM, Normal appearing white matter.
