# Supplementary figures and images for: Insights from DCE-MRI: blood–brain barrier permeability in the context of MS relapses and methylprednisolone treatment
Source: Front Neurosci. 2025 Mar 20;19:1546236. doi: 10.3389/fnins.2025.1546236 (PMC11966965; doi:10.3389/fnins.2025.1546236)

Patlak Ki in NAWM (ml/100g/min)

.1000  
.0800  
.0600  
.0400  
.0200  
.0000  
-.0200

None

One or more

Contrast enhancing lesions

13

11

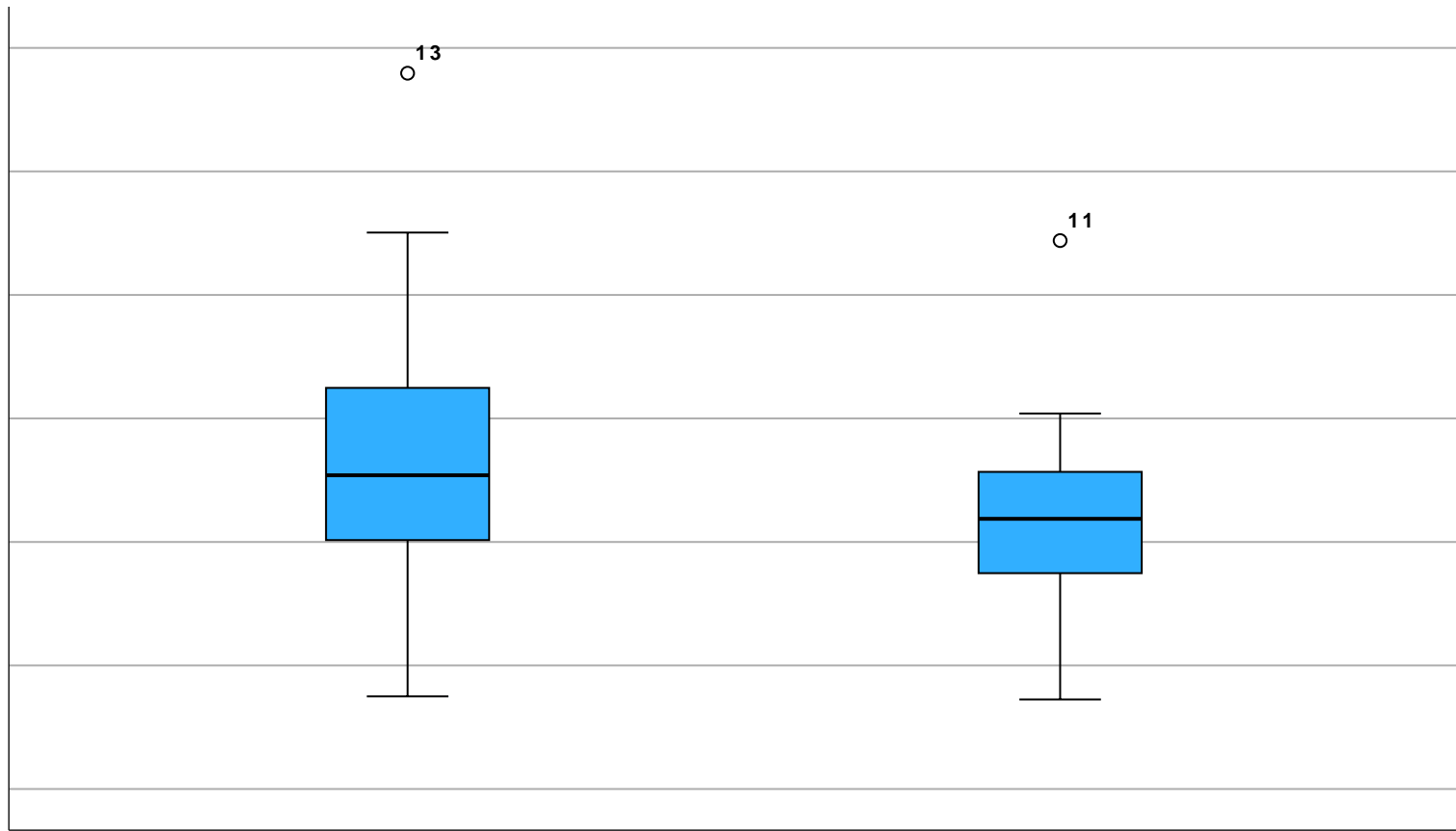

Supplement: SUPPLEMENTARY FIGURE 1 — DCE-MRI overview. Dynamic contrast-enhanced MRI at 3T; 5 slices; 8 mm slice thickness; time resolution 1.25 s, total scan time 15 minutes. [file Data_Sheet_1.pdf]

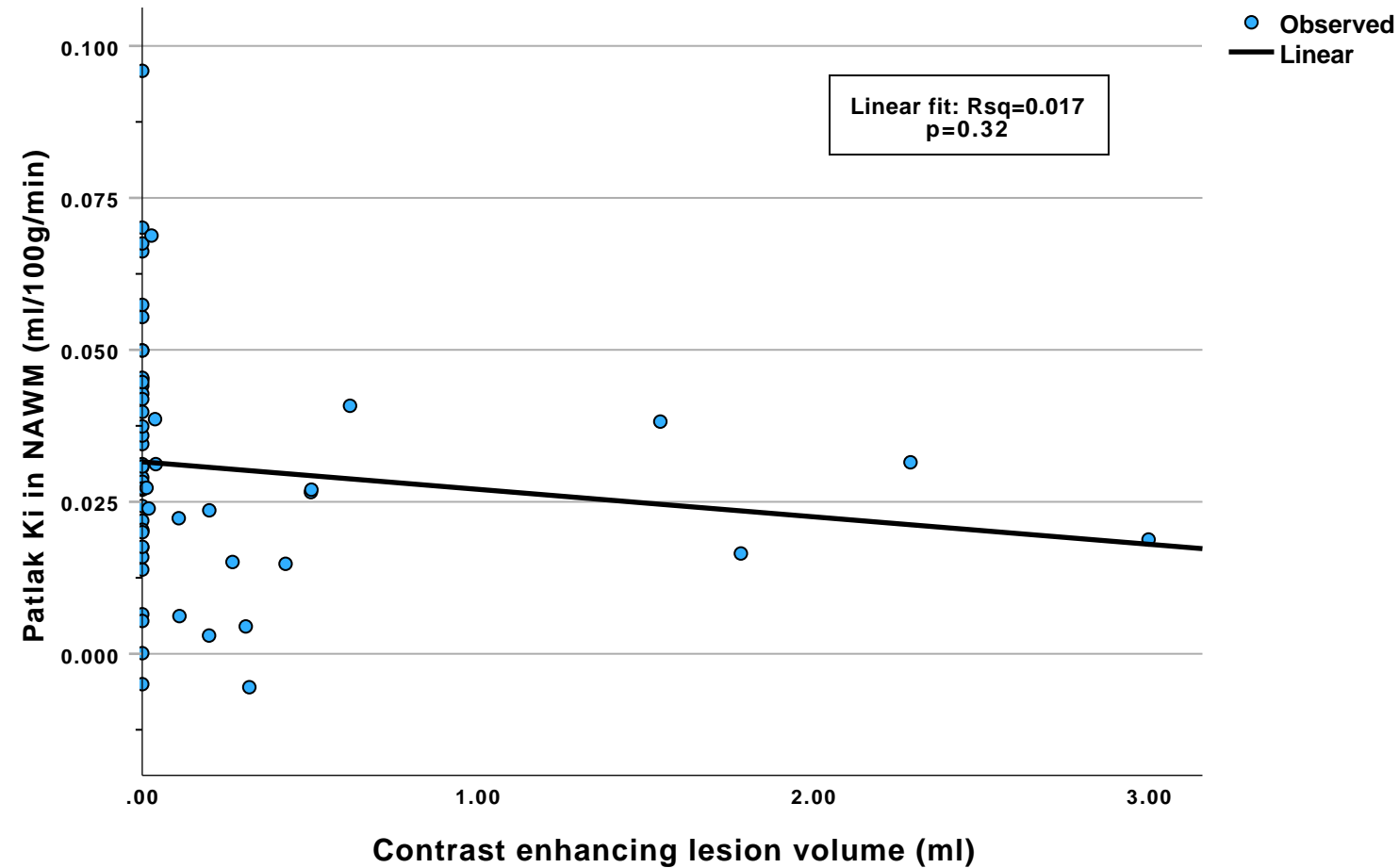

Supplement: SUPPLEMENTARY FIGURE 2 — Ki according to presence on one or more contrast enhancing lesion. No difference in Ki in NAWM was observed in subjects with visually contrast enhancing lesions elsewhere in the cerebrum. NAWM, Normal appearing white matter. [file Data_Sheet_2.pdf]

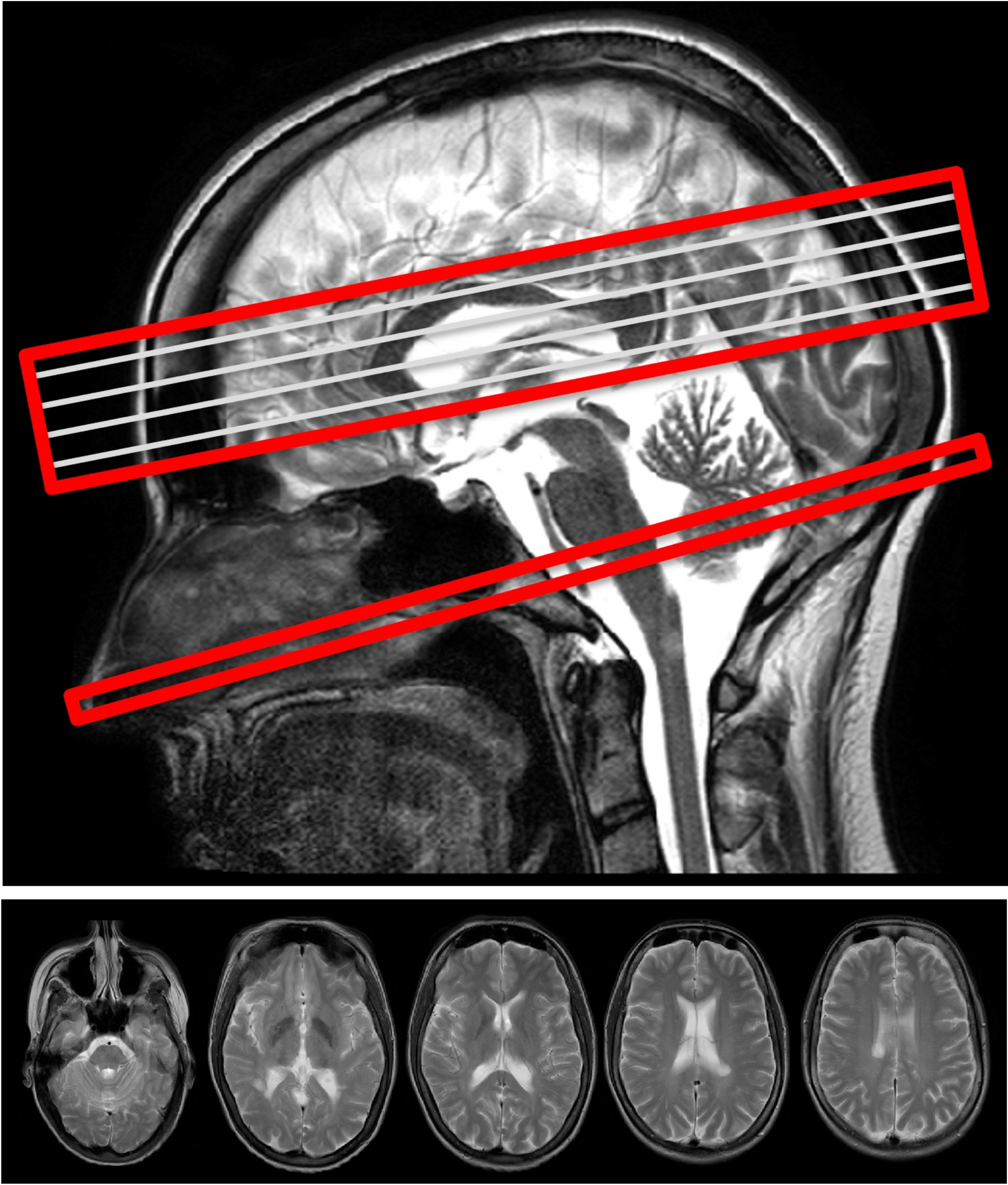

Supplement: SUPPLEMENTARY FIGURE 3 — Ki versus total contrast enhancing lesions volume. Patlak Ki in NAWM plotted against the total volume of visual contrast enhancing lesions in ml. MP, Methylprednisolone; NAWM, Normal appearing white matter. [file Image_1.tiff]
